# Insights into CD24 and Exosome Physiology and Potential Role in View of Recent Advances in COVID-19 Therapeutics: A Narrative Review

**DOI:** 10.3390/life12101472

**Published:** 2022-09-21

**Authors:** Georgios Tsioulos, Ioannis Grigoropoulos, Charalampos D. Moschopoulos, Shiran Shapira, Garyfallia Poulakou, Anastasia Antoniadou, Dimitrios Boumpas, Nadir Arber, Sotirios Tsiodras

**Affiliations:** 1 4th Department of Internal Medicine, Medical School, University General Hospital Attikon, National and Kapodistrian University of Athens, 12462 Athens, Greece; 2 Integrated Cancer Prevention Center, Tel Aviv Medical Center, Tel Aviv 6423906, Israel; 3 3rd Department of Internal Medicine, Medical School, Sotiria General Hospital, National and Kapodistrian University of Athens, 11527 Athens, Greece

**Keywords:** COVID-19, exosomes, CD24

## Abstract

Cluster of differentiation (CD) 24, a long-known protein with multifaceted functions, has gained attention as a possible treatment for Coronavirus Disease 19 (COVID-19) due to its known anti-inflammatory action. Extracellular vesicles (EVs), such as exosomes and microvesicles, may serve as candidate drug delivery platforms for novel therapeutic approaches in COVID-19 and various other diseases due to their unique characteristics. In the current review, we describe the physiology of CD24 and EVs and try to elucidate their role, both independently and as a combination, in COVID-19 therapeutics. CD24 may act as an important immune regulator in diseases with complex physiologies characterized by excessive inflammation. Very recent data outline a possible therapeutic role not only in COVID-19 but also in other similar disease states, e.g., acute respiratory distress syndrome (ARDS) and sepsis where immune dysregulation plays a key pathophysiologic role. On the other hand, CD24, as well as other therapeutic molecules, can be administered with the use of exosomes, exploiting their unique characteristics to create a novel drug delivery platform as outlined in recent clinical efforts. The implications for human therapeutics in general are huge with regard to pharmacodynamics, pharmacokinetics, safety, and efficacy that will be further elucidated in future randomized controlled trials (RCTs).

## 1. Introduction

Coronavirus Disease 19 (COVID-19) still poses a health and socioeconomical burden around the world [1]. The evolution of the virus and the emergence of new variants creates an uncertainty with regard to medical countermeasures. Thus, novel therapeutic agents meeting high efficacy and safety standards, are still required.

Cluster of differentiation (CD) 24 is a long-known protein with multiple functions. It has already been studied in various contexts, such as in cancer diagnosis and prognosis, [2,3] as well as for the treatment of bacterial and viral infections, and sepsis [4]. During the pandemic, CD24 has gained attention as a possible treatment for COVID-19 due to its known anti-inflammatory action [5].

Exosomes and microvesicles are extracellular vesicles (EVs) with an important role in cell-to-cell communication [6]. They share unique characteristics which make them candidates for novel therapeutic approaches in COVID-19 and various other diseases. Due to their ability to carry cargo-molecules, such as nucleic acids and proteins, EVs are considered ideal drug delivery platforms.

The main objective of the current narrative review is to describe the physiology of CD24 and EVs and to present data on their potential role, both independently and combined, in COVID-19 therapeutics.

## 2. *CD24* Gene, Structure, Ligands, and Pathophysiologic Role

CD24 was firstly identified as the heat stable antigen in 1978 by Springer et al., who discovered a heat-resistant protein while investigating unknown cell surface molecules on mice hematopoietic cells [7]. CD24 is a highly glycosylated cell surface protein that is connected with the cell membrane via a glycosyl-phosphatidylinositol (GPI) anchor. The *CD24* gene in humans, identified in 1991 [8], is located on chromosome 6q21, though there are four more loci on chromosomes 1, 15, 20, and Y that represent intron-less pseudogenes [9]. *CD24* is widely expressed in diverse cell types, and produces a small protein with varying molecular weights due to differential glycosylation pattern [3]. The gene transcript consists of an open reading frame of 0.24 kb and an unusually long 3′ UTR (untranslated region) serving as an mRNA stability factor [10]. The protein core contains multiple potential glycosylation sites as well as additional serine and threonine residues, resulting in an adhesion molecule with mucin-like properties [11]. The human precursor protein consists of 80 amino acid residues and is cleaved into a mature peptide of 31 amino acids [12]. Through a cleavage process to remove the N-terminal signal peptide and the C-terminal GPI attachment signal, the preassembled GPI-anchor will be attached to the protein in the endoplasmic reticulum [13]. Additional post-translational modification with N- and O-linked glycosylation gives rise to a glycoprotein with molecular weight ranging from 20 to 75 kDa [3]. Regarding its secondary and tertiary structure, CD24 is thought to be an intrinsically disordered protein, meaning that it does not form a unique structure to exert its function, but instead retains a flexibility which allows different patterns of glycosylation and function [12]. CD24 is highly expressed in immature progenitor cells, as well as in activated cells of the immune system, but minimally in terminally differentiated cells, therefore it has been linked to cell maturation and development [12,14]. Expression of CD24 in hematopoietic cells, such as immature B cells, T cells, granulocytes, dendritic cells, and macrophages is associated with immune cell maturation and regulation of immune responses. However, the molecule has also been identified in non-immune cell types, including neural cells, regenerating muscle cells, and various epithelial cells, as well as in tumors.

CD24 exerts its multifaceted function through the interaction of its incorporated glycans with a variety of ligands. With 14 O- and two N-glycosylation sites, there is a wide repertoire of different cell-specific CD24 glycoforms engaging in cis (same cell) or trans (adjacent cell) interactions [10]. Among the most studied ligands that bind CD24 are P-, L-, and E-selectin, the sialic acid binding immunoglobulin-like lectin (Siglec) 10, the neural cell recognition molecule L1 (or L1CAM), transient axonal glycoprotein (TAG)-1, contactin, and certain damage-associated molecular patterns (DAMPs), such as High Mobility Group Box 1 (HMGB1), heat shock protein (HSP) 70, and HSP90 [15]. CD24, Siglec 10, and HMGB1 form a trimolecular complex that may discriminate pathogen-associated molecular patterns (PAMPs) from DAMPs, and thus infection from tissue injury, respectively [15,16]. The CD24/Siglec 10 interaction has been investigated as a negative regulator of inflammation in sepsis, given that it inhibits Toll-like receptor (TLR) 4-induced cytokine production, and increases the regulatory interleukin (IL)-10 [17]. Using a cecal ligation and puncture (CLP) model to induce lethal sepsis in mice, investigators have suggested that bacterial sialidases may exacerbate sepsis through desialylation of CD24 and disruption of CD24/Siglec immunoregulatory pathway [4].

CD24, as a GPI-anchored protein, lacks a cytoplasmic domain, and it has to interact with other signal transducers in glycolipid-enriched membrane domains or lipid rafts in order to transduce intracellular signaling. Lipid rafts are specialized microdomains of the plasma membrane which contain glycosphingolipids, sphingomyelin, cholesterol, and GPI-anchored proteins, and serve as platforms for membrane signaling and trafficking [14,18]. In this milieu, CD24 interacts with the Src family tyrosine kinases, which in turn regulate, among others, the signal transducer and activator of transcription 3 (STAT3), the mitogen activated protein kinase (MAPK), and the tissue factor pathway inhibitor 2 (TFPI-2) signaling [2,10].

## 3. CD24 Role in Cancer

CD24 overexpression has been observed in a variety of cancer cell lineages, such as in breast [19], ovarian [20] and prostate [21] cancer, bladder [22], lung [23,24] and hepatocellular [25] carcinoma, and non-Hodgkin B cell lymphoma [26], and is considered a significant marker for cancer diagnosis and prognosis [2,3]. In contrast to normal cells, where CD24 is primarily located in the external surface of the plasma membrane, cancerous cells also exhibit cytoplasmic CD24 accumulation, which has been associated with tumor progression, lymph node positivity, and patient survival [13,27]. Moreover, surface CD24 in cancer cells interacts with P- and E-selectins on activated endothelial cells and platelets, promoting rolling and translocation of malignant cells, leading to metastasis [2,28]. Sialyl-Lewis^x^, an abundant sialylated epitope on cancer cell CD24, is an important factor in P-selectin binding and is implicated in breast, ovarian, and lung cell metastasis [13,29]. In cancer cell lines, CD24 also activates β1 integrin, which in turn promotes cell adhesion to extracellular matrix, migration, and metastasis [2,30]. Another important ligand of CD24 for tumor progression is Siglec 10, which is located, among others, on tumor-associated macrophages. CD24/Siglec 10 interaction protects tumor cells from phagocytosis, as it transmits a “don’t eat me” signal [31]. Therapeutic CD24 blockade with monoclonal antibodies has been studied in vitro and in vivo animal models that showed increased phagocytosis of cancer cells, tumor reduction, and improved survival [13,31,32].

## 4. CD24 Role in Immune Response to Pathogens

**Figure 1:**
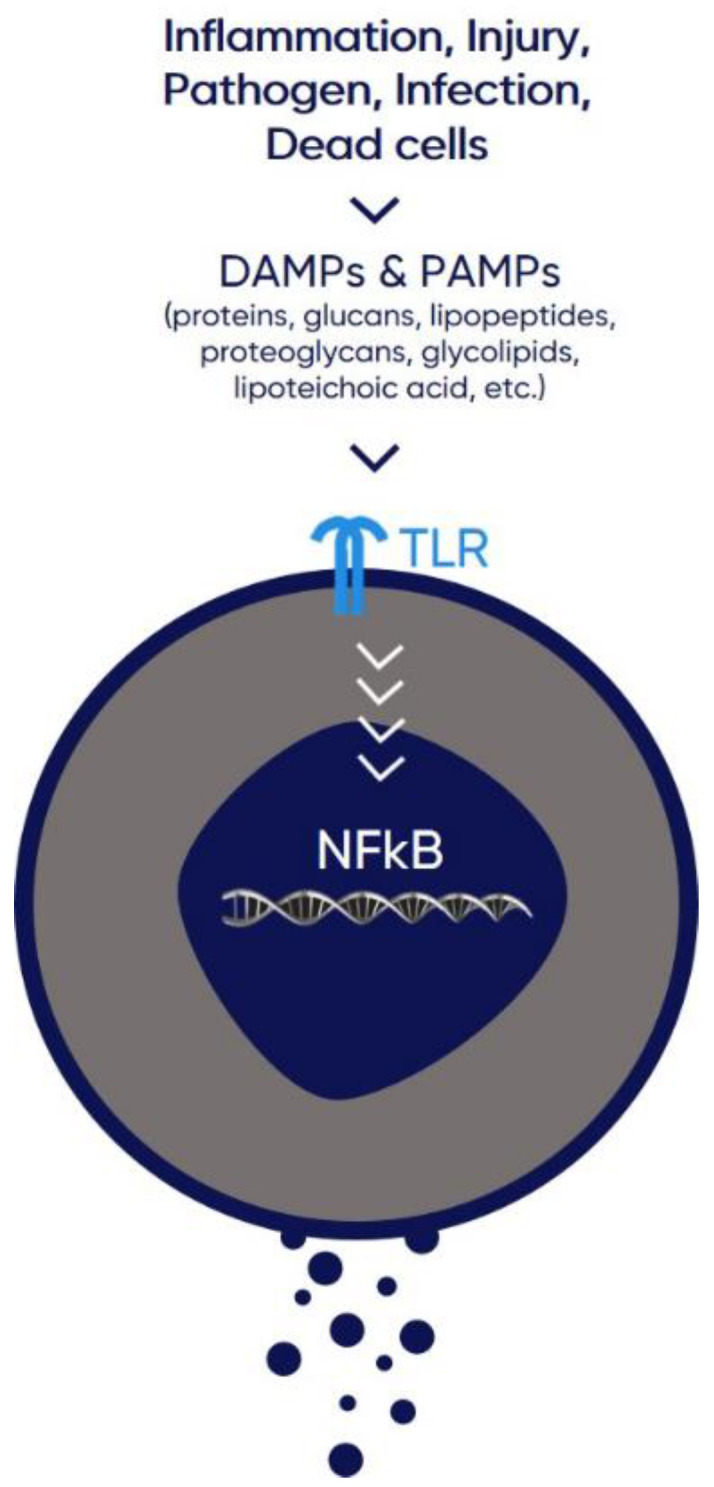
Toll-like receptors (TLRs) on the surface of immune cells, recognize pathogen-associated molecular patterns (PAMPs) or components of injured cells, referred to as damage-associated molecular patterns (DAMPs). TLR metabolic pathway leads to upregulation of nuclear factor-κΒ (NF-κΒ) and proinflammatory cytokine production. Inhibition of NF-κB pathway and consequent cytokine storm, seems to be a promising therapeutic strategy against deterioration of patients with moderate to severe COVID-19.

As mentioned above, CD24 is expressed in a variety of hematopoietic cells, such as B and T cells, and myeloid cells, i.e., neutrophils, eosinophils, dendritic cells, and macrophages [3]. Generally, immature progenitor cells and activated immune cells show high levels of CD24 expression, whereas terminally differentiated populations, such as plasma cells and peripheral T cells, exhibit minimal if any CD24 expression. CD24 plays an important role in B cell maturation, CD28^+^-independent T cell clonal expansion during antigen presentation, as well as T cell homeostatic proliferation. In response to tissue injury, as detected by the presence of DAMPs, CD24 on dendritic and other immune cells bind HMGB1 and form a ternary complex with Siglec 10. The complex elicits inhibitory signals towards the TLR metabolic pathway, which in turn leads to downregulation of nuclear factor-κΒ (NF-κΒ) and proinflammatory cytokine production [17] (Figure 1). Sialidase, a potent virulent factor of various viral and bacterial pathogens, cleaves sialic acid residues from CD24 glycans disrupting its interaction with Siglec 10 and leading to uninhibited inflammatory response. Chen et al. have shown that CD24 or Siglec G (the Siglec 10 murine homolog) gene mutation exacerbates polymicrobial sepsis in mice, whereas administration of sialidase inhibitors leads to reduced sepsis mortality [4]. In contrast with its inhibitory role in innate immunity, CD24 on antigen presenting cells (APCs) is a co-stimulator for CD4 and CD8 T cells. CD24 does not interact with PAMPs and therefore should not hamper antigen-specific immune responses against invading pathogens [13].

## 5. CD24 Role in Autoimmunity

The important role of CD24 in the pathogenesis of autoimmune disease was initially investigated in animal models of experimental autoimmune encephalitis (EAE). Bai et al. found that CD24 deficient mice did not develop EAE, supporting its role as an immune checkpoint. Consequently, the investigators administered in mice with EAE a recombinant fusion protein, binding the extracellular domain of CD24 with human immunoglobin G1 Fc, which activated Siglec G and inhibited the inflammatory response, slowing down the EAE progression [33]. CD24-Fc is currently being studied as a potential therapeutic in conditions characterized by systemic inflammation, as diverse as graft-versus-host disease (GVHD) [34], cancer [13], and COVID-19 [35]. *CD24* gene polymorphisms have also been implicated in the pathogenesis and progression of several autoimmune diseases. A single-nucleotide polymorphism (SNP) in the *CD24* gene (226C to T, rs8734) results in substitution of alanine by valine at residue 57 (A57V) in the GPI-anchor cleavage site, leading to increased expression of CD24 protein [3,13]. CD24^V/V^ has been associated with increased risk for systemic lupus erythematosus (SLE), multiple sclerosis (MS), inflammatory bowel disease, rheumatoid arthritis (RA), and autoimmune thyroiditis [14,36]. On the other hand, a deletion of two nucleotides (TG) at residue 1527 in the 3′-UTR that reduces the CD24 mRNA stability, reduces the incidence of SLE and MS [14,37].

## 6. Potential Use of CD24 in COVID-19

Therapeutic administration of CD24 could potentially be used in disease states characterized by excessive inflammation and immune hyperactivation. The ongoing COVID-19 pandemic presents an opportunity to exploit CD24 use in an appropriate pathophysiologic context as outlined below.

### 6.1. COVID-19 Immunopathology

Pathogenesis of clinically significant COVID-19 features come from the combination of viral replication and immune hyperactivation [38,39], which is often referred to as immune dysregulation [40,41,42]. Products of this extreme inflammatory response can be found in both the lungs and peripheral blood [43]. In summary, these include a wide range of immune cells, such as lymphocytes, monocytes, and neutrophils, and a broad spectrum of cytokines and chemokines. In general, cytokine production is induced when pattern recognition receptors (PRRs), such as TLRs, recognize PAMPs or components of injured cells, referred to as DAMPs, such as HMGB1 and HSP70/90. Moreover, in critical COVID-19 associated illness there is excessive production of cytokines, known as cytokine storm, that drives a multisystemic immune dysregulation that is also known as ‘viral sepsis’ [43,44,45,46]. The cytokine storm is associated with the development of acute respiratory distress syndrome (ARDS), and further clinical deterioration. More specifically, it has been shown that severe cases are characterized by markedly higher levels of IL-2R, IL-6, IL-10, and tumor necrosis factor a (TNF-α) [47] and that elevated levels of IL-6, IL-8, and TNFα are related to increased mortality [48]. It is of great interest that, in contrast to other proinflammatory cytokines, production of interferon-λ (IFN-λ) and type I IFN is reduced and delayed, and induced only in a group of patients deteriorating to critical COVID-19 [49].

### 6.2. COVID-19 and Current Main Therapeutic Strategies

The ever-increasing data concerning the immunopathology of COVID-19, as well as the constantly emerging viral mutations, set an unprecedented challenge in COVID-19 therapeutics.

Despite their proven clinical efficacy, orally administered antivirals such as nirmatrelvir/ritonavir and molnupiravir, are not without drawbacks, mainly related to their mechanism of action and viral evolution, the need for early administration, the varying efficacy in immunocompromised individuals, the presence of contra-indications (e.g., nirmatrelvir/ritonavir cannot be administered in end-stage-renal-disease patients), numerous drug-drug interactions and their availability and cost [50,51]. The intravenous antiviral remdesivir has been shown to diminish time to clinical recovery in hospitalized patients with moderate to severe COVID-19 [52,53,54], despite the fact that not all trials have demonstrated a significant survival benefit [55]. Monoclonal antibodies, even though not clearly beneficial for hospitalized patients [56], are gradually losing their clinical significance as new variants of SARS-CoV-2 emerge, while two of them, bamlanivimab and etesevimab, are no longer recommended against the Omicron variant B.1.1.529. However, bebtelovimab retains action for the currently circulating BA.4 and BA.5 Omicron subvariants [57,58].

Another approach for COVID-19 treatment includes agents that target the abnormal immune activation and hyperinflammation. In this regard, there are three main categories of either available or developing therapeutic strategies: treatments focusing on IFNs, treatments against abnormal inflammatory responses, and those that exploit other pathways [40]. Among them, treatments targeting abnormal inflammatory responses are probably the most important. As previously mentioned, there is a well-established negative correlation between the levels of some of the most important proinflammatory cytokines and severity of COVID-19. Therefore, prevention of the cytokine storm through cytokine neutralization may be significant in preventing deterioration of the disease. The first example of this type of treatment modality is dexamethasone due to its effectiveness against hospitalized patients with respiratory failure [59]. The results of this study paved the way for several treatments targeting specific cytokine receptors, including IL-1 and IL-6, even though not all studies reported a clinical benefit [60,61,62]. Other important members of this category are the Janus kinase inhibitors [63,64]. Among them, baricitinib showed promising results in hospitalized patients, especially those receiving high flow oxygen or noninvasive ventilation [65,66].

### 6.3. CD24 as a Potential Treatment for COVID-19 and Other Syndromes of Immune Activation

As discussed earlier, CD24 functions as a dominant innate immune checkpoint surveillance molecule, that serves as a “don’t eat me” signal [67,68]. CD24 interacts with both DAMPs and Siglec 10. CD24’s link to DAMPs prevents them from binding to the TLRs, therefore inhibiting the NF-ĸB pathway, an extremely significant signaling pathway, that induces the production of cytokines and chemokines [15,31]. At the same time, the CD24-Siglec 10 axis negatively regulates the activity of NF-ĸB through immunoreceptor tyrosine-based inhibition motif (ITIM) domains associated with SHP-1 [SRC homology 2 (SH2)-domain-containing protein tyrosine phosphatase 1] [16,31]. While CD24 interacts with DAMPs and Siglec 10, it does not affect PAMPs’ immune recognition, thereby allowing the innate immune response to achieve viral clearance. It is of interest that a selective downregulation of the Siglec 10 has been found in the damaged lungs of patients with COVID-19 [69]. This data implies a possible clinical benefit after enhancement of the CD24-Siglec 10 pathway in patients with COVID-19.

A recombinant fusion protein composed of the extracellular domain of soluble CD24 linked to a human immunoglobulin G1 (IgG1) Fc domain, named CD24Fc, has been identified as a potential immune checkpoint inhibitor with anti-inflammatory properties. Experimental studies suggest that CD24Fc could diminish the risk and mitigate GVHD in patients undergoing hematopoietic stem cell transplantation [34,70]. In addition, CD24Fc administration to Chinese rhesus macaques with simian immunodeficiency virus (SIV) infection, ameliorated typical acquired immune deficiency syndrome (AIDS) clinical symptoms and, more importantly, decreased AIDS related morbidity and mortality. These encouraging data suggested that CD24Fc could be a potential therapeutic option for human immunodeficiency virus 1 (HIV-1)/AIDS [71]. More recently, CD24Fc injections were found to protect SIV-infected Chinese rhesus monkeys against viral pneumonia and its progression to ARDS [72]. Phase I and phase II clinical trials have evaluated the safety and efficacy of CD24Fc, but further investigation is warranted for COVID-19 patients (NCT02650895, NCT02663622).

Welker et al. conducted a randomized, double-blind, placebo-controlled, phase III study of intravenous CD24Fc versus placebo in hospitalized adults with confirmed COVID-19 and respiratory failure (defined as oxygen saturation < 94%) at nine medical centers in the USA [35]. During a period of five months, 234 eligible participants were randomly assigned (1:1) to receive a single intravenous infusion of either CD24Fc or placebo. All patients received the assigned treatment on top of the standard of care. Time to clinical improvement was the primary endpoint [35]. Patients receiving CD24Fc showed an accelerated time to clinical improvement compared to those in the placebo group over the study period (HR = 1.40, 95% CI 1.02–1.92; *p* = 0.037). This difference was greater among subgroups with less severe COVID-19, suggesting a benefit with earlier administration [35]. Treatment-related adverse events were absent in the CD24Fc group and there was no difference concerning the type and incidence of adverse and severe adverse events between the two groups. This study showed that intravenous CD24Fc could be a promising treatment option for hospitalized patients with COVID-19, especially when administered in the initial stages of the disease [35].

In the context of an extensive correlative analysis, Song et al. studied peripheral blood samples obtained from 22 patients enrolled at a single institution of the above-mentioned study [73] aiming to detect the influence of soluble CD24Fc to immune function and homeostasis of patients with COVID-19 by measuring the activation status of CD8^+^ T-, CD4^+^ T-, and natural killer (NK) cells and levels of an extended spectrum of cytokines and chemokines by using high dimensional spectral flow cytometry. The authors subsequently conducted a network-level analysis for cytokine/chemokine inter-relations [73]. In contrast to the placebo group, a notable reduction in activation status over time for CD8^+^T, CD4^+^T, and NK cells was observed in the CD24Fc group. There was also a reduction in plasma cells with a corresponding increase in mature B cells, a finding that probably suggests either CD24Fc-mediated blockade of peripheral B cells’ differentiation or an accelerated immune clearance of SARS-CoV-2 [73]. Concerning cytokine levels, a fast and prolonged decrease was observed in the CD24Fc group based on a combined cytokine score (expression levels of 30 cytokines were measured). The selective suppression of the host’s immune response by CD24Fc might be a reliable option against the COVID-19 associated hyperinflammatory response, independent of the emerging viral mutations and viral evolution. Nevertheless other mechanisms of actions of CD24Fc may contribute to the therapeutic benefit e.g., accelerated viral clearance due to IL-10 significant downregulation at the CD24Fc treated group, since IL-10 is reported to be connected to enhanced viral replication of HIV, HBV, and HCV [73,74].

### 6.4. CD24 Therapeutic Use in COVID-19 Via Nebulized Exosome Administration

#### 6.4.1. Extracellular Vesicles: General Considerations

EVs are nanosized vesicles derived from cells and play a role in cell-to-cell communication. EVs where first discovered nearly 40 years ago by two separate groups of researchers studying transferrin recycling by reticulocytes [75,76]. Since then, it was gradually revealed that a variety of cells produce EVs [77]. They have also been detected in human plasma [78], urine [79], breast milk [80], and many other body fluids [81].

EVs are membrane-contained vesicles which are secreted by cells and carry DNA, RNA, and proteins [82]. They are classified in three main categories based on their origin and function: apoptotic bodies, microvesicles, and exosomes. Apoptotic bodies vary in size (50–5000 nm) and are secreted during cell apoptosis. Microvesicles are plasma membrane-budded vesicles containing various types of RNAs (mRNA, miRNA, and other non-coding RNA), cytoplasmic and membrane proteins. Their size ranges from 50–1000 nm [6]. Exosomes origin from the endolysosomal pathway and are generally smaller than microvesicles (40–120 nm). Exosomes’ cargo consists of RNA, cytoplasmic and membrane proteins including major histocompatibility complex (MHC) molecules, that microvesicles lack. Due to their different origin, exosomes and microvesicles have some heterogenicity in their membrane composition and cargos. However, because of several uncertainties in biological aspects and absence of a universal purification protocol, it is difficult to completely identify and distinguish exosomes from microvesicles. Thus, these terms are often used interchangeably [6].

Although shortly after their discovery EVs were considered to be the unneeded compounds of a cell [83], ongoing research soon revealed their functional role in cell-to-cell communication, such as antigen presentation by B-lymphocytes [84]. In the past three decades, a plethora of signals mediated by EVs between cells was recognized. For example, it was shown that human cancer cell-derived microvesicles carrying cross-linked tissue transglutaminase and fibronectin can induce mitogenic signals and transform normal fibroblasts [85], while embryonic stem cells shed microvesicles which stimulate trophoblast migration into the uterus [86]. Various mechanisms of action have been described for EVs upon meeting the recipient cell: antigen presentation, transfer of MHC molecules, activating cell’s membrane receptors by linking of carried ligands, transfer of surface receptors and delivery of proteins and nucleic acids inside the recipient cell through membrane fusion or phagocytosis [6,87]. By transferring mRNA, non-coding RNA, and effector proteins, EVs can regulate the epigenome and transcriptome of the recipient cell. Through these mechanisms, exosomes and microvesicles play an important role in physiology and homeostasis, as well as in the pathogenesis of various diseases.

In aging and senescence, senescent cells transmit signals via EVs, which induce expression of senescence-associated secretory phenotype (SASP) factors, such as IL 1-β and tumor growth factor in neighboring cells and thus promote aging in autocrine and paracrine pathways [88,89].

In the lungs, various stimuli such as smoking, hypoxia, and other stressors promote the release of EVs from macrophages, neutrophils, epithelial, endothelial, smooth muscle, and mesenchymal cells. These EVs contain molecules that induce emphysema, production of inflammatory cytokines and fibrosis, leading to development of diseases such as chronic obstructive pulmonary disease (COPD), lung cancer, pulmonary hypertension, asthma, and ARDS [90].

The role of EVs in the cardiovascular system is pleiotropic and may be beneficial or harmful depending on the circumstances under which they are released. Under hypoxic conditions, cardiomyocytes shed exosomes that can induce angiogenesis and neovascularization whereas endothelial cells produce EVs which reduce cardiomyocyte metabolic activity through posttranscriptional gene silencing [91]. On the other hand, after myocardial infarction, cardiomyocyte and endothelial cell-derived EVs promoted the inflammatory response of monocytes infiltrating the infarcted myocardium [92]. Furthermore, under conditions of pressure overload, cardiomyocyte-derived EVs have been shown to enter the bloodstream and transport regulatory signals to remote cells [93,94].

In autoimmune rheumatic diseases, EVs may play a key role in pathogenesis. In patients with SLE, immune complexes containing EVs have been described and are associated with autoantibodies and complement activation. Although the exact cellular origin of these EVs is unknown, they can possibly play a key role in immune responses and subsequent inflammation [95,96]. Additionally, higher levels of EVs have been shown in the synovial fluid of RA patients compared to healthy controls. In particular, platelet-derived EVs are associated with disease severity [97].

Many neurodegenerative diseases share a common mechanism: accumulation of misfolded protein leading to cell damage. The misfolded proteins can spread to specific brain regions, suggesting cellular communication. It is possible that EVs serve as the carriers of the impaired protein [98]. For example, in Alzheimer disease, the tau protein spreads via tau-containing exosomes [99]. It has been shown that inhibition of EVs’ synthesis reduces tau propagation in a mouse model [100].

#### 6.4.2. Exosomes as a Novel Delivery Platform for Therapeutics

In the last decade, exosomes and microvesicles have gained considerable scientific attention as therapeutic agents due to their structural and functional properties, relating to multiple systems and diseases [101]. The structural similarity of EVs to the membrane of cells from which they originate gives them the advantage of high biocompatibility and low immunogenicity [102]. Additionally, they are able to adhere to cells, permeate tissues, and even cross blood-brain barrier via protein membrane-compounds that interact with integrins [103]. Alongside with their ability to carry molecules-cargos such as proteins and nucleic acids, exosomes and microvesicles are unique candidates for drug delivery platforms.

Engineered EVs are natural EVs modified to carry proteins for cell-targeting purposes, loaded with therapeutic agents or isolated from parental cells genetically engineered to produce specific RNA sequences or proteins. Before EVs can be widely available for clinical research, there are some drawbacks that need to be addressed [103,104]. Firstly, many aspects of the structure and function of EVs still remain unclear. Given the wide variety of physiological procedures they mediate, it is impossible to predict what the side effects of EVs in clinical practice might be. Secondly, there is no universal protocol for EV manufacturing. This is a major issue for comparable results in clinical trials when applying EVs in human diseases therapeutics. To address that, the International Society of Extracellular Vesicles (ISEV) published the “Minimal information for studies of extracellular vesicles (MISEV)” position statement in 2014 (updated in 2018), regarding isolation and characterization procedures of EVs [105,106]. Lastly, although many important steps have been made in the past decades, large scale production remains a problem. Currently, a variety of isolation techniques for large scale production exists, such as ultracentrifugation, ultrafiltration, and size exclusion chromatography among others. However, some limitations remain due to relatively high cost and time-consuming methods [107].

#### 6.4.3. Exosomes in COVID-19

Early in the SARS-CoV-2 pandemic, Rao et al. described a possible therapeutic intervention of EVs in COVID-19 disease [108]. Engineered EVs displaying abundant ACE-2 and cytokine receptors demonstrated effectiveness in preventing SARS-CoV-2 infection of human colorectal adenocarcinoma epithelial Caco-2 cells in vitro via antagonistic binding of the virus to EVs’ ACE-2 receptors. Additionally, the same EVs showed promising in vitro results in neutralizing inflammatory cytokines. In an acute lung injury (ALI) mouse model, intratracheal administration of the engineered EVs suppressed lung injury in a dose-dependent manner without any signals of systemic toxicity [108].

Only few human trials of exosomes for COVID-19 treatment have been published so far. Sengupta et al. administered mesenchymal stem cell (MSC)-derived exosomes intravenously to 24 patients with severe COVID-19 pneumonia in a non-randomized open-label clinical trial. This study showed promising results regarding reversal of hypoxia and downregulation of cytokine storm. Importantly, no significant adverse events were noted [109]. Zhu et al. evaluated the use of nebulized allogenic adipose tissue mesenchymal stromal cell-derived exosomes in a phase IIa single-arm clinical trial of 7 severe COVID-19 patients. Similarly to the previous trial, no prespecified adverse events were reported [110]. Lastly, Chu et al. published a pilot trial of nebulized MSC-derived exosomes in 7 patients with COVID-19 showing no safety signals [111].

**Figure 2:**
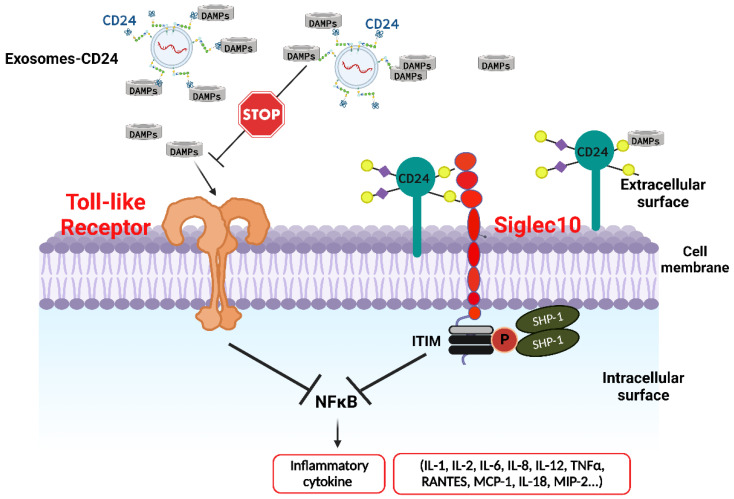
Exosomes carrying CD24, named EXO-CD24, combine advantages of both exosomes as a novel drug delivery platform and CD24 as a potent immune checkpoint surveillance molecule. CD24 interacts with both DAMPs and Siglec 10. CD24’s link to DAMPs prevents them from binding to the TLRs, therefore inhibiting the NF-ĸB pathway that induces the production of cytokines and chemokines. At the same time, the CD24-Siglec 10 axis negatively regulates the activity of NF-ĸB through ITIM domains associated with SHP-1. While CD24 interacts with DAMPs and Siglec 10, it does not affect immune recognition through PAMPs, thereby allowing the innate immune response to achieve viral clearance. CD24, Cluster of differentiation 24; DAMPs, damage-associated molecular patterns; ITIM, immunoreceptor tyrosine-based inhibition motif; NF-κΒ, nuclear factor-κΒ; PAMPs, pathogen-associated molecular patterns; Siglec 10, sialic acid binding immunoglobulin-like lectin 10; SHP-1, SRC homology 2-domain-containing protein tyrosine phosphatase 1; TLR, Toll-like receptor. Created with BioRender.com (accessed on 7 September 2022).

A novel approach for COVID-19 therapeutics was introduced by Shapira et al., who implemented exosomes enriched with CD24, named EXO-CD24 [5] (Figure 2). In a preceding in vitro phase, investigators reported inhibition in cytokine and chemokine production by EXO-CD24-treated human monocytes that were previously stimulated by phorbol 12-myristate 13-acetate (PMA) [5]. Subsequently, in vivo studies in mice that used the murine analogue of CD24, EXO-mCD24, reported no adverse events, suggesting possible safety of the medication in humans. Possible efficacy of EXO-mCD24 was supported by data coming from two experimental models in mice. More specifically, inhaled EXO-mCD24 diminished cytokine production and inflammation-related lung injury, in lipopolysaccharide (LPS)-induced ARDS mouse models, and improved survival in cecum ligation and puncture (CLP)-induced abdominal sepsis models [5].

The phase Ib/IIa clinical trial that followed demonstrated safety and potential efficacy in 35 humans with COVID-19 pneumonia when EXO-CD24 was administrated via inhalation. Safety was the primary endpoint of the specific clinical trial [5]. Thirty-five participants with COVID-19 pneumonia were enrolled to inhale EXO-CD24 once daily for five consecutive days divided into four dose escalation groups. There was no drug-related adverse event reported over the study period of 35 days or during the follow-up period in none of the groups. In terms of efficacy, although safe conclusions are blunted by the absence of a placebo group, there were some promising results. Those included amelioration of symptoms and other clinical parameters, such as oxygen saturation, respiratory rate, time to discharge, improvement of lung infiltrates in chest radiographs or CTs, and reduction of blood inflammatory markers and cytokines [5]. Without any doubt, the administration of EXO-CD24 in patients with COVID-19 showed encouraging results in terms of safety and efficacy. The study is followed by two clinical trials, one that was recently completed in 91 patients with moderate to severe COVID-19 in 2021 in our centers in Greece (ClinicalTrials.gov Identifier: NCT04902183; results under preparation) and a larger phase IIb placebo-controlled randomized clinical trial (RCT) (ClinicalTrials.gov Identifier: NCT04969172; currently recruiting) (Table 1).

## 7. Conclusions

In conclusion, the presented data support the fact that CD24 has an important physiological role in inflammation and immune surveillance without hampering antigen-specific immune responses against invading pathogens. Furthermore, it may act as an important immune regulator in diseases with complex physiologies characterized by hyperinflammation. Pilot studies depict significant amelioration of the inflammatory milieu in the setting of mild to moderate COVID-19 treated by CD24 administration. In this regard, the potential therapeutic use of CD24 in other conditions, where immune dysregulation plays a key pathophysiological role e.g., ARDS and sepsis, should be further exploited. Additionally, CD24—and, in the same manner, other therapeutic molecules—can be administered with the use of exosomes exploiting their unique characteristics to create a novel drug delivery platform. The implications for human therapeutics in general are significant with regard to pharmacodynamics, pharmacokinetics, safety, and efficacy that will be further elucidated in future RCTs.

## Figures and Tables

**Table 1:** Indicative animal and human studies investigating CD24 as a therapeutic target.

Pre-Clinical Studies				
Animal	CD24 Formulation	Disease/Condition	Mechanism	Outcome
Rhesus macaques	CD24-Fc	SIV	targets DAMP-induced chronic inflammation	decreased AIDS morbidity and mortality [71]
Mice	CD24-Fc	GVHD	Siglec-G/CD24 axis targeting	enhancement of Siglec-G/CD24 interaction controls GVHD severity [34]
Mice	anti-CD24 mAb + Pseudomonas exotoxin	Colorectal cancer	antibody-drug conjugate	tumor reduction (78%) [112]
Mice	anti-CD24 mAb + nitric oxide	Hepatocellular carcinoma	antibody-drug conjugate	tumor inhibition (81%) [113]
Mice	CD24Fc	Rheumatoid arthritis	targets DAMP-induced cytokine production	TNF-a, IL-6, MCP-1, IL-1ß reduction, reduced inflammatory cell infiltration in joint synovium [114]
Diet-induced obese mice	CD24Fc	Obesity, insulin resistance and NASH	targets chronic inflammation of metabolic syndrome	reduction in weight, lipids, glucose, insulin resistance, steatosis, hepatic fibrosis [115]
**Human clinical trials**				
**Trial ID**	**CD24 formulation**	**Disease/Condition**	**Mechanism**	**Outcome**
NCT02663622 NCT04095858	CD24-Fc	GVHD prevention		Terminated
NCT04317040	CD24-Fc	COVID-19	targets inflammation in response to tissue injury	accelerates clinical improvement of hospitalised patients with COVID-19 who are receiving oxygen [35]
NCT04747574 NCT04902183 NCT04969172	EXO-CD24 (inhaled)	COVID-19	cytokine storm suppression	Safety and possible efficacy (NCT04747574) Results under preparation (NCT04902183) Currently recruiting (NCT04969172)

Abbreviations: AIDS, acquired immune deficiency syndrome; COVID-19, coronavirus disease 2019; DAMP, damage-associated molecular pattern; GVHD, graft-versus-host disease; IL, interleukin; mAb, monoclonal antibody; NASH, non-alcoholic steatohepatitis; SIV, simian immunodeficiency virus; TNF-a, tumor necrosis factor alpha.
